# Supplementary material for: Mamba neural operator: Who wins? transformers vs. state-space models for PDEs
Source: J Comput Phys. Author manuscript; Available in PMC 2026 Oct 16. (PMC7619586; doi:10.1016/j.jcp.2025.114567)
Supplement: Supplementary file 1 [file EMS218482-supplement-Appendix.pdf]

Appendix A. Notation table

To improve readability and address any ambiguity, we summarise in Table A.5 the notation used throughout the paper. The table groups symbols by context (Mamba Neural Operator, General PDE and hyperparameters) and provides a concise description of their meaning. This helps ensure consistency across sections and serves as a quick reference for the reader.

Appendix B. More explanation on the main method

In this section, we provides an additional example on how the difference between Euler and ZOH method. Consider the scalar linear state–space model

$$h'(t) = ah(t) + bu(t), \tag{B.1}$$

Table A.5
Comprehensive notation reference for symbols used throughout the manuscript. The table provides quick lookup for mathematical definitions and aligns notation across different sections.

Notation	Description
$h(t) \in \mathbb{R}^N$	State vector at continuous time t
$u(t) \in \mathbb{R}^L$	Control/input vector
$y(t) \in \mathbb{R}^L$	Output vector
$A \in \mathbb{C}^{N\times N}$	State transition matrix
$B \in \mathbb{C}^N$	Input-to-state matrix
$C \in \mathbb{C}^N$	State-to-output matrix
$D \in \mathbb{C}^N$	Direct input-to-output matrix
Δ	Time discretisation step size
$x_k \approx x(t_k)$	State at discrete time index k, with $t_k = k\Delta$
$u_k \approx u(t_k)$	Input at discrete time index k
$y_k \approx y(t_k)$	Output at discrete time index k
$A_d = e^{A\Delta}$	Discretised state transition matrix (from matrix exponential)
$B_d = \int_0^\Delta e^{A\tau} B\, d\tau$	Discretised input matrix
$x_{k+1} = A_d x_k + B_d u_k$	Discrete-time state update equation
$y_k = Cx_k + Du_k$	Discrete-time observation equation
Hyperparameters	
d_state	size of the SSM hidden state
ssm_ratio	share of d_{model} channels in the SSM branch

with $a = -1$, $b = 2$. We look at one step of size $\Delta > 0$ from t_k to $t_{k+1} = t_k + \Delta$. For the Forward Euler, we defines as follows:

$$h_{k+1}^{\text{Euler}} = (1 + a\Delta)\, h_k + b\Delta\, u_k. \tag{B.2}$$

For the Zero-Order Hold (ZOH), we defines as follows:

$$h_{k+1}^{\text{ZOH}} = e^{a\Delta}\, h_k + \left(\int_0^\Delta e^{a\tau}\, d\tau\right) bu_k = e^{a\Delta}\, h_k + \frac{e^{a\Delta} - 1}{a}\, bu_k, \tag{B.3}$$

which corresponds to the matrix pair $(\tilde{A}, \tilde{B}) = \left(e^{A\Delta},\ A^{-1}(e^{A\Delta} - I)B\right)$ in the scalar case.

If we truncate the exponential at first order,

$$e^{a\Delta} \approx 1 + a\Delta, \qquad \frac{e^{a\Delta} - 1}{a} \approx \Delta, \tag{B.4}$$

we recover the Euler pair $(I + \Delta A, \Delta B)$.

If we Let $u(t) \equiv 1$ on $[t_k, t_{k+1})$ with arbitrary h_k.

$$h_{k+1}^{\text{ZOH}} = e^{-\Delta} h_k + 2(1 - e^{-\Delta}),$$
$$h_{k+1}^{\text{Euler}} = (1 - \Delta)h_k + 2\Delta.$$

Then Using Talyr expansion, we obtain

$$\underbrace{h_{k+1}^{\text{ZOH}}}_{\text{exact}} = \left(1 - \Delta + \tfrac{\Delta^2}{2} - \cdots\right)h_k + 2\left(\Delta - \tfrac{\Delta^2}{2} + \tfrac{\Delta^3}{6} - \cdots\right). \tag{B.5}$$

Euler keeps only the first-order pieces: $h_{k+1}^{\text{Euler}} = (1 - \Delta)h_k + 2\Delta$. Then the per-step difference is $h_{k+1}^{\text{ZOH}} - h_{k+1}^{\text{Euler}} = \frac{\Delta^2}{2}\, h_k - \Delta^2 + O(\Delta^3) = O(\Delta^2)$.

B.0.0.1. Numerical illustration (from $h_k = 0$)..

- $\Delta = 0.5$: $h_{k+1}^{\text{ZOH}} = 2(1 - e^{-0.5}) \approx 0.78694$, $h_{k+1}^{\text{Euler}} = 1.0$, error ≈ 0.21306.
- $\Delta = 0.25$: $h_{k+1}^{\text{ZOH}} \approx 0.44240$, $h_{k+1}^{\text{Euler}} = 0.5$, error ≈ 0.05760.

As Δ halves, the error shrinks by a factor of ≈ 4 (consistent with $O(\Delta^2)$ local error), while ZOH is exact under the constant-input assumption.

Appendix C. More time analysis results

Consistent with the main section, we provide additional long-time stability analysis on a different dataset with an alternative baseline. Fig. C.11 illustrates the long-time integration stability of OFormer and Galerkin Transformer, both with and without the incorporation of our proposed MNO. The OFormer baseline demonstrates a noticeably faster convergence rate during training compared to our proposed model. This behavior can be attributed to OFormer's normalization-based operator formulation, which effectively

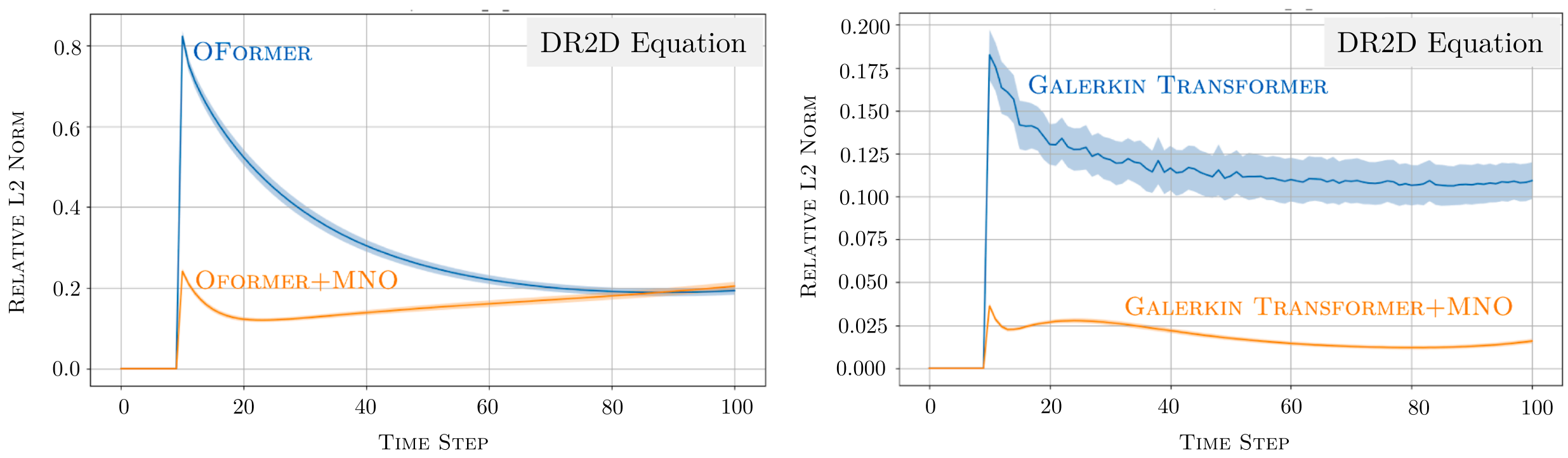

Fig. C.11. Relative L2 error on DR2D across time steps, comparing the performance of our Mamba Neural Operator (MNO) with the OFormer baseline and the Galerkin Transformer (G.T.). The left plot shows the evolution of relative L2 error over time steps for the DR2D equation, while the right plot shows the same for the DR2D equation. MNO consistently outperforms OFormer and G.T., particularly in terms of lower error growth, highlighting its superior ability to capture long-range dependencies and model complex dynamics more effectively.

stabilizes the optimization dynamics by rescaling both the input and operator spectra. Such normalization reduces gradient variance and facilitates smoother propagation through layers, thereby accelerating convergence. Although OFormer exhibits a steeper early decrease in relative L2 error after the initial spike (faster short-horizon convergence), our proposed Mamba neural operator suppress that spike and maintain lower error growth over the rollout. For the OFormer backbone the asymptotic errors are comparable, but MNO avoids the large early overshoot and yields a smoother, more stable trajectory. In both cases, the baseline models (blue curves) exhibit rapid error growth during extended rollout, with the error plateauing at a relatively high level or even drifting upward. By contrast, when leveraging Mamba neural operator (orange curves), the models maintain substantially lower error throughout the entire temporal horizon. The error remains bounded and exhibits minimal drift, indicating that MNO effectively mitigates the accumulation of integration errors over long rollouts. This demonstrates that our method not only improves short-term predictive accuracy but also provides enhanced numerical stability and robustness for long-time forecasting of dynamical systems.
